# Comparative effects of intranasal and intraperitoneal resveratrol on the eye–brain axis in a cisplatin-induced neurotoxic rat model

**DOI:** 10.1038/s41598-026-48629-6

**Published:** 2026-04-29

**Authors:** Ghadha Ibrahim Fouad, Hanan F. Aly, Mohamed I. Mabrouk, Mohamed B. Shalaby, Karima A. Hamed, Wagdy K. B. Khalil, Maha Z. Rizk

**Affiliations:** 1 Department of Therapeutic Chemistry, Pharmaceutical and Drug Industries Research Institute, National Research Centre, 33 El-Bohouth St, Dokki, Cairo, 12622 Egypt; 2 Department of Physical Therapy, Faculty of Applied Medical Sciences, Applied Science Private University, Amman, Jordan; 3 Toxicology Research Department, Research Institute of Medical Entomology (RIME), General Organization of Teaching Hospitals and Institutes (GOTHI), Ministry of Health and Population (MoHP), Dokki, Cairo, 12311, Egypt; 4 Department of Cell Biology, National Research Centre, 12262 El-Bohouth St, Cairo, Egypt

**Keywords:** Cisplatin, Intranasal, Resveratrol, Retinal neurotoxicity, DNA damage, IL-10, 8-OHdG, Biochemistry, Cancer, Drug discovery, Medical research, Neurology, Neuroscience

## Abstract

Cisplatin (CISP) is a platinum-based chemotherapeutic drugs that are commonly used to treat several types of cancers. However, its clinical application faces several hurdles that could limit its clinical efficiency such as central and retinal neurotoxicity. Hence, it is crucial to develop antioxidant-based co-therapeutic approaches to mitigate CISP-induced neurotoxicity. Resveratrol (RSV) is a naturally occurring polyphenol that exhibits a plethora of health-promoting activities. The present study aimed to compare the neuroprotective impact of intranasal (IN) and intraperitoneal (IP) doses of RSV on the alleviation of CISP-induced neurotoxicity. Rats were allocated into four groups: Negative control, CISP-rats, treated CISP-rats with IN RSV, and treated CISP-rats with IP RSV. Assessment of lipid peroxides and catalase activities in the brain and ocular tissues were conducted. The brain acetylcholinesterase (AChE) activity and the eye 8-Hydroxy-2-deoxyguanosine (8-OHdG) content were estimated. Furthermore, the gene expression of interleukin-10 (IL-10) and caspase-8 was performed. In addition, serum nuclear factor erythroid 2-related factor 2 (Nrf2) level, as well as the extent of DNA damage in blood cells were evaluated. Histopathological analyses of different regions of the brain and the eye tissues were conducted. Our findings demonstrated that both IN and IP doses of RSV mitigated the central and retinal toxicity as demonstrated biochemically and histopathologically; indicating the anti-oxidant, anti-inflammatory, genoprotective, and anti-apoptotic activities of RSV. Collectively, IP RSV exhibited a superior cumulative neuroprotective activity than IN RSV. However, IN administration of RSV could be regarded as a simple and alternative non-invasive therapeutic approach for receiving RSV with further improvements.

## Introduction

Cisplatin (CISP) is one of the most potent platinum-based antineoplastic agents with a tremendously effective mechanism of pharmacological action. Several studies are warranted to delineate its efficacy in combination therapy to improve its therapeutic outcome, by using natural and synthetic antioxidants, to mitigate CISP-associated oxidative stress, systemic toxicity, and metabolic alterations^1^^,2^. Despite its poor ability to cross the blood-brain barrier (BBB), CISP is capable of stimulating morphological alterations in the white matter structure and disrupting neurotransmitters^3^. CISP-induced neurotoxicity might trigger neuroinflammation due to the ability of the pro-inflammatory cytokines, to cross BBB, induce neuroinflammatory responses, and disrupt the BBB’s integrity and function^4^.

The retina is considered an anatomical and developmental extension of the central nervous system (CNS)^5^^,6^, and its vascular system also has morphologically and physiologically similar properties to the cerebrum^7^. As an extension of the brain, the retina has similar characteristics with the brain, and also retina could be regarded as a potential diagnostic tool for neurodegenerative disorders, thereby several studies explored the link between the retinal alterations and neurodegeneration^8^^,9^. For example, Alzheimer’s disease is characterized by BBB disruption^10^; similarly, the neural loss and apoptosis of peripheral cells in the retinal vascular system could result in the thinning and dilation of blood vessels, leading to “Retinal venous beading” which is a chronic dilation of the retinal vein after retinal ischemia or other abnormalities^11^, and can result in different clinical manifestations, such as hard exudates, venous occlusion, retinal neovascularization and leakage^12^.

Toxic effects of CISP on the retinal and corneal tissues have been previously shown experimentally and clinically, which may cause vision loss^13,16^. Activation of microglia, which is the primary resident immune cell in the brain and retina, results in the release of inflammatory mediators and generation of reactive oxygen species (ROS)^17^. Retinal excitotoxicity is suggested as a major mechanism of neurodegeneration, and experimental models of excitotoxicity are extensively used for studying retinal ganglion cells (RGCs) damage and dysfunction in ocular diseases, including optic neuropathy^18,20^. In addition, the retina can be imaged noninvasively, providing a unique way for the detection of the status of the brain. Therefore, it is necessary to investigate whether retinal abnormalities are associated with neurodegeneration, which will provide new insights for the early diagnosis and treatment of neurodegenerative disorders including chemotherapeutic drugs-induced neurotoxicity.

CISP, like many other chemotherapeutic agents, exerts its neurotoxic potential mainly through inducing oxidative stress and DNA damage, which can trigger cellular and endothelial senescence; leading to BBB disruption and vascular dysfunction^21,24^. Chemotherapeutic drugs trigger oxidative DNA damage in endothelial cells, leading to an irreversible cell cycle arrest and cause profound endothelial dysfunction that could be manifested as loss of endothelial integrity, increased permeability, alterations in the extracellular matrix and secretion of pro-inflammatory and inflammatory cytokines, all of which contribute to BBB disruption and vascular dysfunction^24^^,25^.

CISP-induced neural damage can activate an inflammatory response, which includes the microglial and astrocytic activation, and finally contributes to neuronal damage and neuroinflammation, thereby exacerbating the neurotoxicity^26^. Interleukin-10 (IL-10) is a classic immunoregulatory and anti-inflammatory cytokine, M2 phenotype of activated microglia can release high levels of anti-inflammatory cytokines, such as IL-10, correlated with neuroprotection, recovery, and repair in various neurodegenerative diseases^27^.

A combination neurotherapeutic approach targeting oxidative stress, endothelial senescence, and inflammatory signaling may be particularly potent in maintaining BBB and other vascular functions, protecting endothelial function, and alleviating the risk of chemotherapy-induced neurotoxicity. Therefore, identifying neuroprotective targeted interventions could improve the quality of life and the neurological outcomes for cancer survivors undergoing CISP-based chemotherapy through alliviating CISP-associted neurotooxicity^24^. Pre-clinical studies have demonstrated that antioxidant therapy can in various neurovascular injury models, suggesting its potential application in chemotherapy-induced BBB and vascular disruption ; therefore, antioxidants could be a promising strategy to protect neurovascular integrity, by preventing ROS-induced damage to tight junction proteins and endothelial cells^24^^,28^.

Resveratrol [3, 5, 4′-trihydroxystilbene] (RSV) is a polyphenolic compound that has 2 forms; *cis*-RSV and *trans*-RSV and it exists as a monomer or as an oligomer containing about 2–4 monomer units, and it is naturally present in grapes, berries, and plums. RSV exhibited anti-oxidative, anti-inflammatory, anti-neoplastic, anti-atherogenic, and anti-angiogenic activities^29^^,30^. When taken orally, RSV is highly absorbed through the gastrointestinal tract and quickly metabolized in the liver and then excreted in the urine; therefore, only a smaller portion of the absorbed RSV will eventually reach the internal organs, rendering its potential dependent on the administration route^31^. Therefore, this study investigated both the intranasal (IN) and intraperitoneal (IP) routes of administration of RSV.

The IN route was used as a safe alternative therapeutic method for the delivery of some therapeutic agents, such as hormone replacement therapy or insulin^32,34^. IN administration bypasses gastrointestinal destruction and hepatic metabolism. Therefore, IN drug delivery route effectively increases drug bioavailability, and enables faster onset of action^35^; intranasally-administered drugs are as effective as one performed by intravenous or rectal administration, and exhibited a better duration of action and efficacy than one administered orally or sub-lingually^36^. In addition, the olfactory route is more efficient for decreasing hepatic and renal clearance and the systemic exposure^37^.

The presence of BBB prevents the entrance of potential therapeutic agents into the brain^38^; therefore, IN route of administration circumvents first-pass metabolism and can facilitate specific accumulation of agents in brain versus peripheral tissue^39^. Accordingly, the respiratory or olfactory biodistribution pathways of drugs from the nasal mucosa to the neural tissues could be regarded as simple, safe, rapid, patient-friendly, non-expensive, non-invasive, and alternative delivery methods that facilitate the passage of drugs through the BBB^4^^,40^. Therefore, the IN route of administration appears as an ideal therapeutic approach for neurodegenerative disorders, that enables the direct delivery of bioactive therapeutic molecules to the brain^41^^,42^; through using direct “nose-to-brain” route, with the administration of the anti-oxidant in specific nasal formulations and its passage to the CNS mainly through the olfactory nerve way^43^.

Eventhough it has been demonstrated that RSV can enter the brain from the blood stream in animals and humans, RSV demonstrated poor distribution in the CNS in comparison to other tissues^44^. In addition, the intensive metabolization of orally-administered RSV strengthens the rationale for its effective IN use; furthermore, IN administration of RSV is expected to increase its transferability to the brain and improve therapeutic efficacy, outcome, and safety^43^.

Consequently, to bridge the current gap, we need to assess whether targeting the eye-brain axis through intranasal administration of RSV offers an advantage over its systemic administration in mitigating CISP-induced central and retinal neurotoxicity; this could be achieved by comparing the neuro-retinal protective potentials of IN and IP administrations of RSV, which may be superior or comparable, by comparing their activities in modulating oxidative stress, inflammation, and apoptosis in CISP-induced neurotoxicity.

## Materials and methods

### Chemicals

CISP vial (50 mg/25 ml injectable solution) was purchased from Mylan S.A.S. (Saint-Priest, France). RSV was purchased from Sigma Chemical Company, St. Louis, USA. All the remaining chemicals and diagnostic kits were procured from Biodiagnostic Company, (Giza, Egypt) and Sigma Chemical Company, St. Louis, USA. Phosphate Buffered Saline (PBS, product number: P2272) was purchased from Sigma-Aldrich Chemicals Company, USA. Thiopental sodium (Thiopental) was sourced from Biochemie GmbH, Austria.

### Experimental animals

Thirty-two female albino Wistar rats; 6–8 weeks old weighing 140 ± 10 g, were acquired from Animal House of the National Research Center (NRC), Egypt. They were acclimatized for one week prior to the experiment, under standard housing of 24 ± 2 °C with a humidity of 45 ± 5% and kept in a 12/12 h light/dark cycle. A standard rat chow and water *ad libitum* were fed to the rats. This study was performed in line with the principles of the Declaration of Helsinki. Approval was granted by the Ethics Committee of the NRC, Egypt (approval *no.* 04410824).

### Dosing RSV

The selected dose 10 mg/kg of RSV was chosen based on doses reported in the literature using rodent models in previous studies^45,47^.

The IN administration of RSV was received according to the previous study^4^; that was conducted using a polyethylene tip attached to a micropipette, and inserted approximately 5 mm into the right nostril without anesthesia. RSV was suspended in phosphate buffer saline (PBS; pH 7.4). The head-back supine positioned rats, with a 70–90° tilt, received the IN dose of RSV gently and slowly into the nasal cavity; to facilitate RSV uptake into the brain. The nostril was observed closely for signs of blockage or irritation. The dose of 10 mg/kg of RSV was determined, where a dose of 25 µl of RSV was received for each rat for 14 consecutive days, as illustrated in Table 1.

### Experimental design

The rats were randomly divided into four groups (*n* = 8) and the experiment proceeded as follows:

Group I Negative Control (NC) group: Rats IN-administrated 25 µl PBS into the right nostril for 14 days, interrupted by two IP doses of distilled water on the 7th and 14th .

Group II CISP group (CISP): CISP-induced rats were given two i.p. dose (7 mg/kg BW) of CISP (on the 7th and 14th days of the experiment) and served as positive control, according to Ibrahim Fouad et al.^4^ and Altunkaya et al.^26^.

Group III CISP group administrated IN RSV (CISP + RSV-IN): treated CISP-neurotoxicated rats were IN inoculated with RSV (25 µl /nostril; daily) for 7 days interrupted by a single dose (7 mg/kg BW; i.p. injection) of CISP, followed by 7 days of IN RSV, and followed by a single i.p. dose (7 mg/kg BW) CISP.

Group VI CISP group administrated IP RSV (CISP + RSV-IP): treated CISP-neurotoxicated rats were administrated IP RSV (10 mg/kg BW; daily) for 14 days interrupted by two doses of CISP (7 mg/kg BW; i.p. injection) separated by 7 days of IP RSV^46^.

**Table 1:** Demonstrating the experimental design and the dosing regimen. Four experimental groups were included: Negative control, CISP-induced control, CISP + RSV-IN, and CISP + RSV-IP. Two i.p. doses (7 mg/kg BW) of CISP were received on the 7th and 14th days of the experiment. The prepared suspension of 10 mg/kg BW of RSV was administrated to the two RSV-treated CISP groups; either IP or IN (25 µl /nostril of RSV).

Experimental group	Category	CISP	RSV	
		IP	IN	IP
Group I	NC	XXX	XXX	XXX
Group II	CISP	Two i.p. doses (7 mg/kg BW) on the 7th and 14th days	XXX	XXX
Group III	CISP + RSV-IN		25 µl /nostril for 14 days	XXX
Group IV	CISP + RSV-IP		XXX	10 mg/kg BW; daily for 14 days

### Sampling and preparation of brain and eye tissues

The rats were sacrificed by cervical dislocation under sodium thiopental anesthesia (50 mg/kg; i.p. dose). The whole-brain tissue was dissected and washed with normal saline. The right half of each brain and each eyeball was weighed and homogenized in 5 ml ice-cold PBS (50 mM, *p*H 7.4) using a glass-Teflon homogenizer, centrifuged at 6000 rpm for 20 min using a high-speed cooling centrifuge, and the clear supernatant obtained after centrifugation was frozen at -20 °C to be utilized for the estimation of biochemical parameters of oxidative stress of lipid peroxide or malondialdehyde (MDA), and catalase (CAT). The left half of each brain sample and each eyeball were fixed at 10% buffered formalin for 24 h and specified for histopathological investigation.

## Biochemical analyses

### In the brain and eye (ocular) tissues

#### Determination of brain and eye contents of lipid peroxides (LPO)

Brain and eye contents of lipid peroxides (LPO) or malondialdehyde (MDA), a measure of lipid peroxidation, were assayed by measuring the thiobarbituric acid reactive substances, according to the method of Ohkawa et al.^48^.

#### Determination of brain and eye contents of catalase (CAT)

Antioxidant catalase (CAT) enzyme activities in the brain and eye tissues were measured spectrophotometrically at 510 nm, according to Aebi^49^. CAT activity was expressed as units/g tissue.

### In the brain tissues

Acetylcholinesterase (AChE) Activity: AChE activity in the brain tissues was determined according to Gorun et al.^50^. The reaction mixture contained 135 µL of distilled water, 20 µL of 100 mM potassium phosphate buffer (pH 7.4), 20 µL of 10 mM DTNB, 5 µL of diluted tissue homogenate (1:10, v/v), and 20 µL of 8 mM acetylthiocholine iodide as a substrate. The absorbance was measured at 412 nm. Results were expressed as µmol/mg protein.

### Gene expression of IL-10 and caspase-8 genes

RNA Isolation: Total RNA was extracted from brain tissues of rats of different experimental groups, using TRIzol reagent (Invitrogen, Germany) according to the manufacturer’s protocol. The RNA samples were treated with 1 U of RQ1 RNase-free DNase (Invitrogen, Germany) to eliminate residual genomic DNA and dissolved in DEPC-treated water. RNA purity was verified spectrophotometrically by the A260/A280 ratio (1.8–2.1), and integrity was confirmed by the presence of distinct 28 S and 18 S rRNA bands on ethidium bromide-stained formaldehyde agarose gels51,52. RNA samples were either used immediately for reverse transcription or stored at -80 °C until use.

Reverse Transcription (RT) Reaction: Complementary DNA (cDNA) was synthesized from total RNA using the RevertAid First Strand cDNA Synthesis Kit (MBI Fermentas, Germany), following the manufacturer’s protocol. Briefly, 5 µg of total RNA was reverse transcribed in a 20 µl reaction mixture containing 5× RT buffer, 50 mM MgCl₂, 10 mM dNTPs, 50 µM oligo(dT) primer, 20 U RNase inhibitor, and 50 U M-MuLV reverse transcriptase. The reaction was performed at 25 °C for 10 min, followed by 42 °C for 60 min, and terminated at 99 °C for 5 min. The resulting cDNA was stored at -20 °C until use in real-time PCR analysis.

*Real-Time Polymerase Chain Reaction (RT-PCR)*: Gene expression levels were quantified using the Step-One Real-Time PCR System (Applied Biosystems, Thermo Fisher Scientific, Waltham, MA, USA). Reactions were performed in a 25 µL total volume containing 12.5 µL of 1× SYBR Premix Ex Taq (TaKaRa, Biotech Company, Ltd.), 0.5 µL of each primer (0.2 µM), 6.5 µL of nuclease-free water, and 5 µL of cDNA template^53,^^54^. The thermal cycling conditions were as follows: initial denaturation at 95 °C for 3 min, followed by 40 cycles of 95 °C for 15 s, 55 °C for 30 s, and 72 °C for 30 s. A final melting curve analysis was performed from 60 °C to 95 °C with 0.5 °C increments every 10 s to confirm amplification specificity. Distilled water was used as a negative control. Primer sequences for each gene are listed in Table 2. Relative gene expression levels were calculated using the 2^⁻ΔΔCT^ method^55^.

**Table 2:** Primers sequence used for *qRT-PCR*. IL-10: interleukin 10; GAPDH: glyceraldehyde-3-phosphate dehydrogenase.

Gene	Primer sequence	NCBI reference
IL-10	F: ata act gca ccc act tcc ca.	NM_012854.2
	R: ttt ctg ggc cat ggt tct ct	
Caspase-8	F: ggt tac agc tct cct acc cc	NM_022277.2
	R: tgt ctt cct cca. aca tcc cc	
GAPDH	F: aag atg gtg aag gtc ggt gt	AF106860.2
	R: tga ctg tgc cgt tga act tg	

### In the eye (ocular) tissue

Measurement of 8-Hydroxy-2-deoxyguanosine (8-OHdG): DNA was extracted from eye tissues by homogenization in a buffer containing 1% sodium dodecyl sulfate, 10 mM Tris-HCl, and 1 mM EDTA (pH 7.4), followed by overnight incubation with proteinase K (0.5 mg/mL) at 55 °C. The homogenates were subsequently treated with RNase (0.1 mg/mL) at 50 °C for 10 min and extracted with chloroform/isoamyl alcohol. DNA was precipitated by adding 3 M sodium acetate and two volumes of absolute ethanol, incubated at -20 °C, washed twice with 70% ethanol, air-dried, and dissolved in 10 mM Tris-1 mM EDTA buffer (pH 7.4). DNA digestion was performed according to Patel et al.^56^. The oxidative DNA adduct 8-OHdG was quantified using high-performance liquid chromatography (HPLC) equipped with a CoulArray detection system (Model 5600). Detection was achieved with electrochemical sensors connected in series and UV monitoring at 260 nm.

### In the blood

Estimation of serum levels of Nrf2: Nrf2, a key transcription factor regulating antioxidant defense, was quantified in serum samples using a sandwich ELISA kit specific for rat Nrf2, according to the manufacturer’s instructions. Briefly, serum samples were added to pre-coated wells with anti-rat Nrf2 antibodies, followed by incubation with biotinylated secondary antibodies and streptavidin-horseradish peroxidase (HRP). The colorimetric reaction was developed and measured at 450 nm, and Nrf2 concentrations were expressed as ng/mg protein^57^.

Comet Assay: The comet assay was conducted according to Blasiak et al.^58^ with minor modifications. Blood samples were mixed with low-melting-point agarose (1:10, v/v) and layered onto slides pre-coated with normal-melting-point agarose. After solidification at 4 °C, a third agarose layer was added and slides were incubated in lysis buffer for 60 min at 4 °C. Slides were then immersed in alkaline unwinding solution for 60 min in the dark, followed by electrophoresis at 0.8 V/cm and 300 mA for 30 min at 4 °C. After neutralization and dehydration in 70% ethanol, slides were stained with ethidium bromide and examined under a Zeiss epifluorescence microscope (excitation 510–560 nm; barrier filter 590 nm) at ×400 magnification. Cells were scored visually on a scale of 0–3 based on tail length and DNA migration^59,]^^60^.

### Histopathological examination

Specimens from brain (cerebral cortex, hippocampus, and cerebellum) and eye (retina and cornea) were collected at the end of the experiment, fixed in neutral buffered formalin 10%, washed, dehydrated, cleared and embedded in paraffin. The paraffin embedded blocks were sectioned at 5µ thickness and stained with hematoxylin and eosin^61^ for histopathological examination.

### Histopathological lesion scoring

Alterations in the brain and eye tissues were recorded and scored as: no changes (0), mild (1), moderate (2) and severe (3) changes, the grading scores were determined by percentage: <30% changes (mild change), < 30% – 50% (moderate change), and > 50% (severe change)^62^.

### Statistical analysis

Results were expressed as mean ± SEM (standard error of the mean) and were analyzed using the Duncan test as a post hoc test with a significance level (*p* < 0.05). Statistical Package for the Social Sciences (SPSS, version 20) origin software was used for all data (URL Link: https://www.ibm.com/products/spss-statistics). The percent of the difference in the value of data for the treated group with respect to the negative control or positive CISP control was also calculated and presented as a percentage difference. Percentage of change % = (treated value − control value) / control value × 100.

## Results

### Impact of route of administration of RSV on oxidative stress in CISP-intoxicated brain and eye tissues

**Fig. 1:**
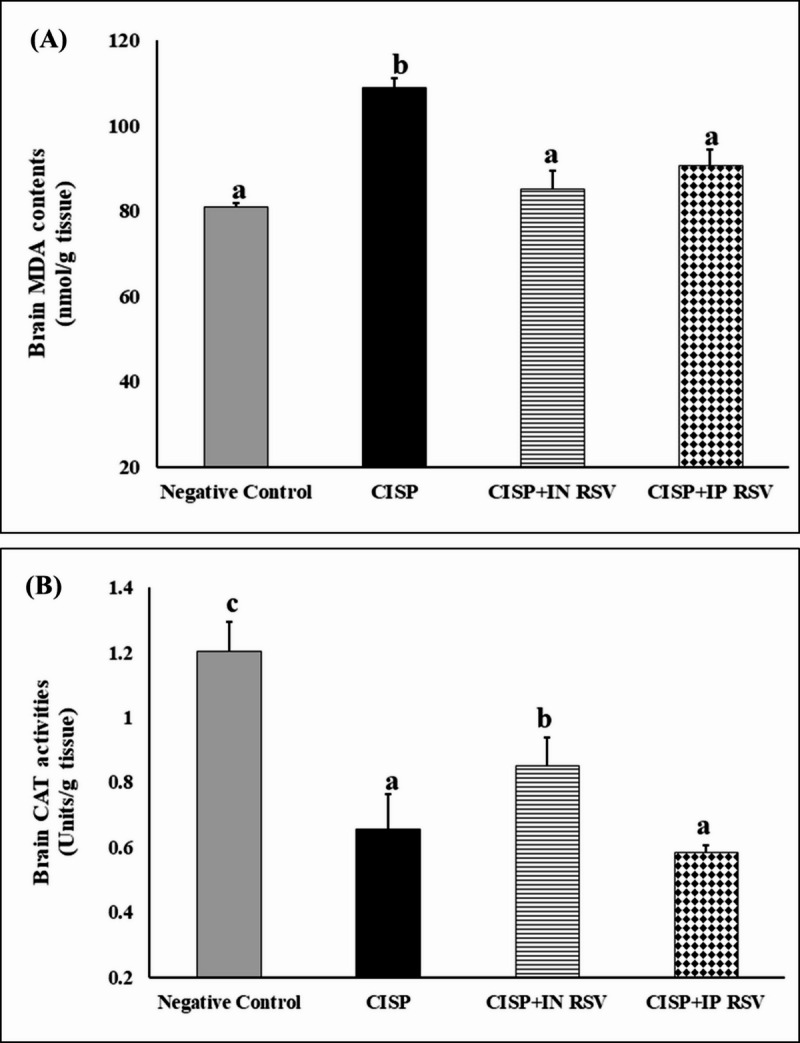
Impact of treatment of CISP-exposed rats with RSV, either IN or IP, on Lipid peroxidation (MDA) and catalase (CAT) activities in the brain tissues of different groups. Data are presented as mean ± SEM. The unlike superscript letters (**a**, **b**, **c**, **d**) indicated that mean values were significantly different at *p* < 0.05.

**Fig. 2:**
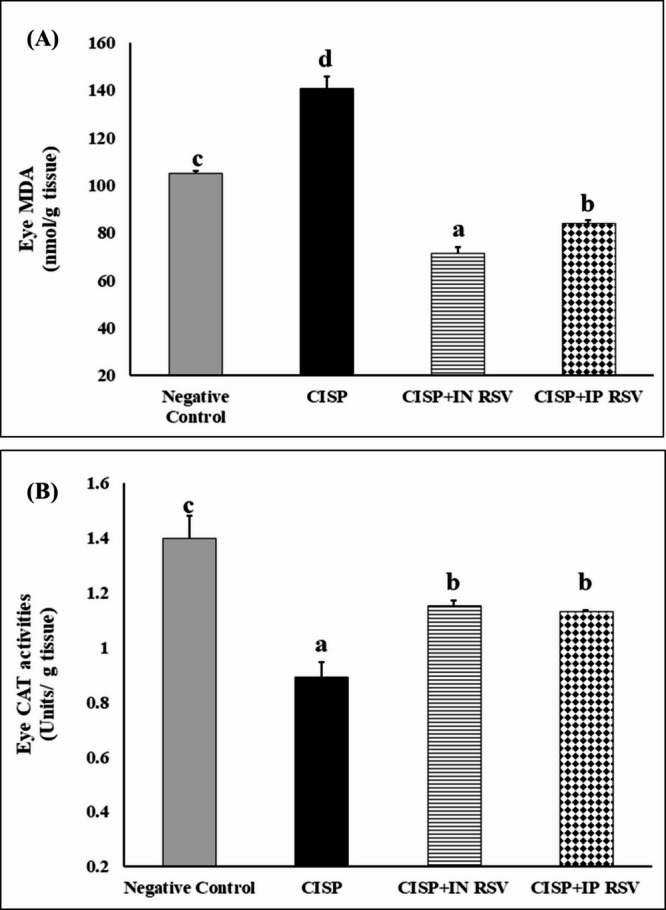
Impact of treatment of CISP-exposed rats with RSV, either IN or IP, on Lipid peroxidation (MDA) and catalase (CAT) activities in the eye tissues of different groups. Data are presented as mean ± SEM. The unlike superscript letters (**a**, **b**, **c**, **d**) indicated that mean values were significantly different at *p* < 0.05.

CISP intoxication caused a significant increment in lipid peroxidation and MDA formation by 34.69 and 34.02%, along with a significant decline in CAT activities by 45.46, and 36.07%, respectively in the brain and the eye tissues of CISP-intoxicated rats, as compared to control rats (Figs. 1 and 2).

On the other hand, treatment of CISP-intoxicated rats with RSV IN or IP elicited a significant reduction in lipid peroxidation (-21.7 and − 16.67%) and (-49.1 and − 40.31%), respectively, in the brain and the eye tissues of CISP-RSV treated rats, as compared to CISP rats.

While CAT was significantly elevated upon treatment of CISP-intoxicated rats with RSV IN or IP (30 and 29.08%) and (10.79 and 26.86%), respectively, in the brain and the eye tissues of CISP-RSV treated rats, as compared to CISP rats.

The results demonstrated that both IN and IP administrations of RSV exhibited a comparable anti-oxidative potential against CISP-induced oxidative stress.

### Impact of route of administration of RSV on generation of 8-OHdG in CISP-intoxicated eye tissues

#### Assessment of 8-OHdG generation

**Fig. 3:**
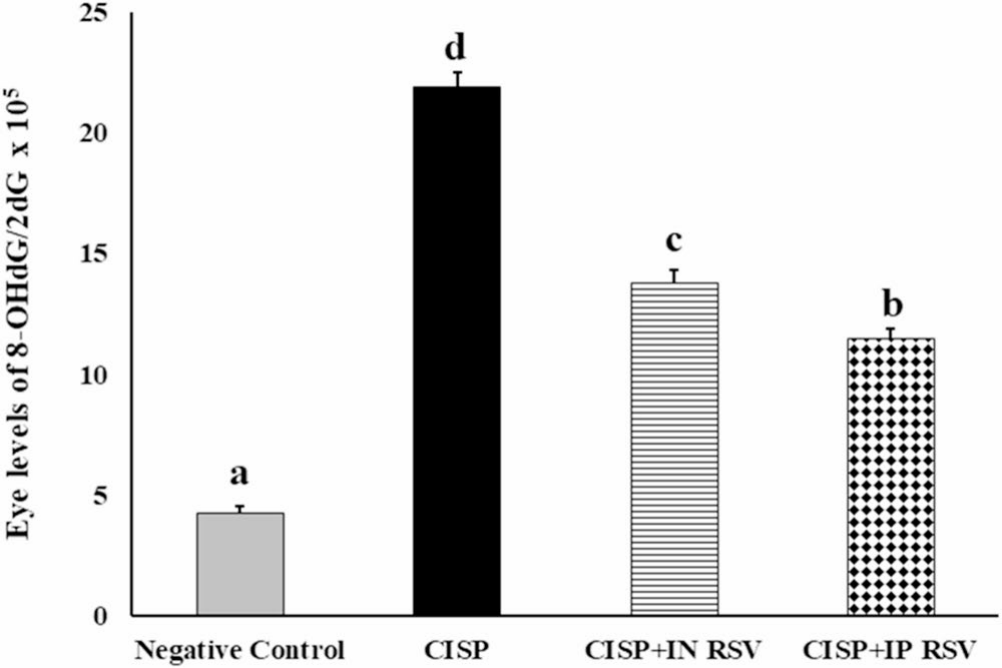
Generation of 8-OHdG in the eye genome of CISP-exposed rats and treated with IN or IP RSV. DNA damage was expressed as the ratio of oxidized DNA base (8-OHdG) to nonoxidized base (2-dG) in eye DNA. Data are presented as mean ± SEM. The unlike superscript letters (**a**, **b**, **c**, **d**) indicated that mean values were significantly different at *p* < 0.05.

The 8-OHdG generation in the eye tissues of rats of different experimental groups following exposure to CISP and treatment with RSV (IN or IP) is summarized in Fig. 3. CISP intoxication resulted in a significant elevation in 8-OHdG generation in the eye tissues of CISP-rats by 414.39%, as compared to negative control rats. On the other side, IN and IP administration of RSV to CISP-rats caused a significant reduction in 8-OHdG generation by -36.78 and − 47.34%, respectively, as compared to CISP rats. The results demonstrated that IP administration of RSV exhibited a superior DNA protective activity, relative to that of IN RSV against CISP-induced generation of 8-OHdG and DNA damage in the ocular tissues.

### Impact of route of administration of RSV on neurochemical changes in CISP-intoxicated brains

#### Inhibition of AChE activity

**Fig. 4:**
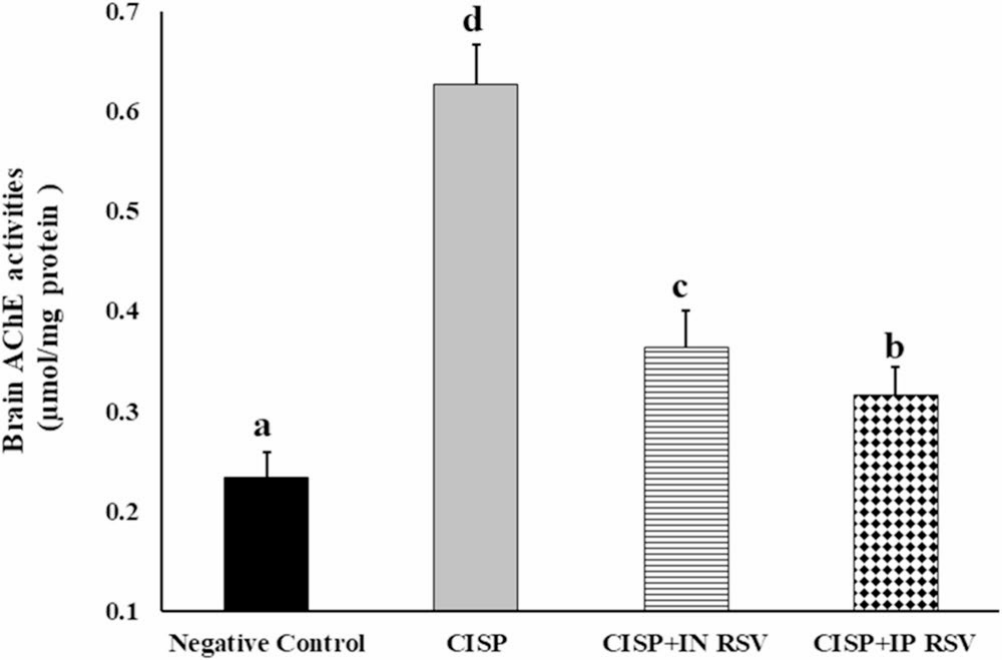
Impact of treatment of CISP-exposed rats with RSV, either IN or IP RSV, on AChE activities in the brain tissues of different groups. Data are presented as mean ± SEM. The unlike superscript letters (**a**, **b**, **c**, **d**) indicated that mean values were significantly different at *p* < 0.05.

Figure 4 shows the activity of AChE in brain tissues of CISP-exposed rats and treated with RSV (IN or IP) in different experimental groups. AChE activity was increased significantly by 169.1% (*P* < 0.01) (0.627 ± 0.04 µmol/mg protein) in CISP-rats versus that in control group (0.233 ± 0.03 µmol/mg protein) (Fig. 4). Treatment of CISP-rats with IN and IP of RSV decreased significantly (*P* < 0.05) the activity of AChE (0.364 ± 0.04 and 0.316 ± 0.03 µmol/mg protein, respectively), by (-42, and − 49.6%, respectively), as compared to CISP-exposed rats (0.627 ± 0.04 µmol/mg protein). The results indicated that IP administration of RSV exhibited a comparable anti-AChE activity to that of IN RSV, against CISP-induced AChE activity in the brain.

### Induction of neuroinflammation (IL-10 gene) and apoptosis (caspase-8 gene)

**Fig. 5:**
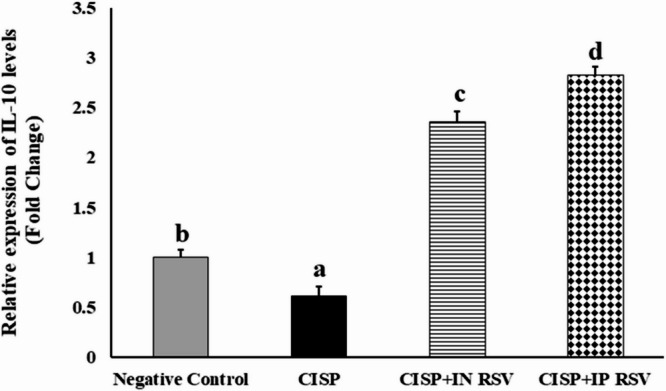
The expression alterations of IL-10 gene in brain tissues of rats exposed to CISP and treated with RSV, either IN or IP, in the brain tissues of rats of different groups. Data are presented as mean ± SEM. The unlike superscript letters (**a**, **b**, **c**, **d**) indicated that mean values were significantly different at *p* < 0.05.

**Fig. 6:**
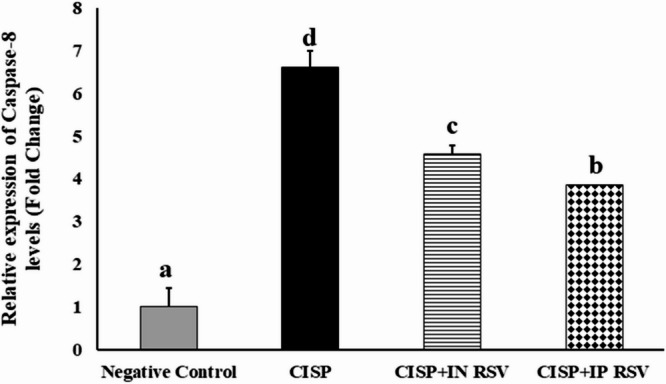
The expression alterations of caspase-8 gene in brain tissues of rats exposed to CISP and treated with RSV, either IN or IP, in the brain tissues of rats of different groups. Data are presented as mean ± SEM. The unlike superscript letters (**a**, **b**, **c**, **d**) indicated that mean values within treatment were significantly different at *p* < 0.05.

The present study explored the gene expression analysis of IL-10 and Caspase-8 genes in the brain tissues of rats exposed to CISP and treated with RSV (either IN or IP) (Figs. 5 and 6).

The results demonstrated the downregulation of the expression levels of IL-10 in CISP rats by 61% (0.61 ± 0.0971-fold change), as compared with control rats (1.00 ± 0.0743-fold change). However, the expression levels of IL-10 gene were upregulated significantly (*P* < 0.001) by 386.9% and 463% in the brain tissues of treated CISP-rats with either IN (2.36 ± 0.1035-fold change) or IP (2.83 ± 0.0840-fold change) doses of RSV, respectively, as compared to CISP-rats (0.61 ± 0.0971-fold change) (Fig. 5). Our findings indicated that IP RSV exhibited a superior potential to modulate neuroinflammation to that of IN RSV, against CISP-induced neuroinflammation in the brain.

On the other hand, the expression levels of caspase-8 were over-expressed by 662% in CISP-exposed rats (6.62 ± 0.440-fold change 1) as compared with control rats (1.00 ± 0.0955-fold change). Nevertheless, the expression levels of caspase-8 gene were downregulated significantly (*P* < 0.01) by 69.3% and 58.3% in brain tissues of CISP-exposed rats treated with IN RSV (4.59 ± 0.376-fold change) and IP RSV (3.86 ± 0.1852-fold change), respectively, compared to those in CISP-rats (6.62 ± 0.4401) only (Fig. 6). Our results showed that IP RSV exhibited a superior potential, to that of IN RSV, to inhibit CISP-induced apoptosis in the CISP-induced brains.

In other words, CISP intoxication caused a significant upregulation in the gene expression of caspase-8 by 562%, along with a significant downregulation in the gene expression of IL-10 by -39%, in CISP-exposed brains, as compared to negative control rats. However, treatment of CISP-rats with RESV either IN or IP demonstrated a significant downregulation in the expression of caspase-8 (-30.66 and − 41.69%), along with a significant upregulation in the expression of IL-10 (286.89 and 363.93%), as compared to CISP-exposed rats. The cumulative findings indicated that IP RSV is more potent then IN RSV against CISP-induced neuroinflammation and apoptosis.

### Impact of route of administration of RSV on the biochemical changes in CISP-intoxicated blood cells

#### Serum Nrf2 levels

**Fig. 7:**
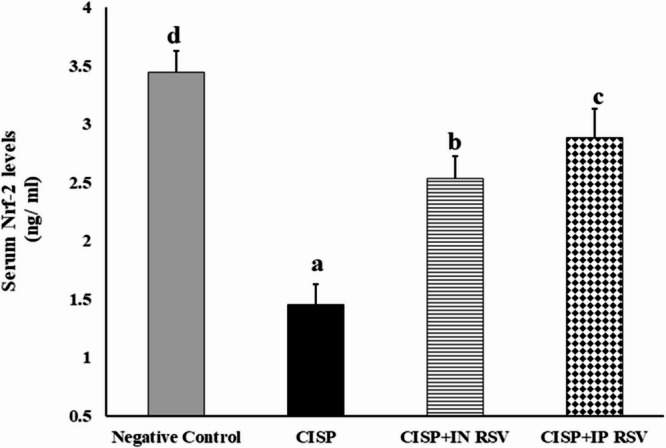
Impact of treatment of CISP-exposed rats with RSV, either IN or IP, on serum Nrf2 levels in rats of different groups. Data are presented as mean ± SEM. The unlike superscript letters (**a**, **b**, **c**, **d**) indicated that mean values were significantly different at *p* < 0.05.

Serum Nrf2 levels in the experimental groups of rats exposed to CISP and treated with RSV (either IN or IP) are shown in Fig. 7. CISP reduced significantly the levels of Nrf2 by 42.2% (*P* < 0.01) (1.453 ± 0.177 ng/ml) as compared that in control group (3.447 ± 0.180 ng/ml) (Fig. 7). Treatment of CISP-rats with either IN or IP RSV increased significantly (*P* < 0.05) the levels of Nrf2 (2.537 ± 0.186 ng/ml and 2.883 ± 0.246 ng/ml, respectively), in comparison to those in CISP-rats (1.453 ± 0.177 ng/ml) only (Fig. 7).

CISP intoxication caused a significant decrement in serum Nrf-2 levels by -57.85% in CISP-exposed rats, as compared to negative control rats. However, treatment of CISP-rats with RESV either IN or IP demonstrated a significant increase in serum Nrf-2 levels by 74.60 and 91.41%, as compared to CISP-exposed rats. Our results showed that IN RSV exhibited a superior potential in increasing serum Nrf-2 levels, to that of IP RSV, to inhibit CISP-induced oxidative stress. This aligns with the previous data concerning the superior antioxidative activities of IN RSV, relative to IP RSV.

#### DNA damage in blood cells

The DNA damage in blood cells of rats exposed to CISP and treated with either IN of IP of RSV is illustrated in Table 3. The results showed that the rates of percentage of DNA damage in blood cells exposed to CISP were increased (23.60 ± 1.778%) significantly (*P* < 0.001) as compared with negative control group (7.40 ± 0.872%). However, the rates of DNA damage in CISP-exposed rats treated with either IN of IP RSV were decreased significantly (*P* < 0.05) (17.20 ± 1.158 and 13.80 ± 1.068%, respectively), as compared with those in CISP-exposed rats (23.60 ± 1.778%) only.

CISP intoxication caused a significant increase in the percentage of DNA damage % in blood cells by 218.92% in CISP-exposed rats, as compared to negative control rats. On the other side, treatment of CISP-rats with RESV either IN or IP demonstrated a significant reduction in DNA damage % in blood cells by -27.12 and − 41.53%, as compared to CISP-exposed rats. Our results showed that IP RSV exhibited a superior potential in mitigating DNA damage % in blood cells, to that of IN RSV. This aligns with the previous data concerning the superior DNA protective activity of IP RSV, relative to IN RSV, against CISP-induced generation of 8-OHdG and DNA damage in the ocular tissues.

**Table 3:** Impact of treatment of CISP-exposed rats with RSV, either IN or IP, on DNA damage in blood cells of rats of different groups. *: Number of cells examined per a group, ^**^: Class 0 = no tail; 1 = tail length < diameter of nucleus; 2 = tail length between 1X and 2X the diameter of nucleus; and 3 = tail length > 2X the diameter of nucleus. Data are presented as mean ± SEM. The unlike superscript letters (a, b, c, d) indicated that mean values were significantly different at *p* < 0.05.

Treatment	No. of cells		Class^**^				DNA damaged cells % (Mean ± SEM)
	Analyzed^*^	Comets	0	1	2	3	
Negative control	500	37	463	33	4	0	7.40 ± 0.872 ^**a**^
CISP	500	118	382	28	41	49	23.60 ± 1.778 ^**d**^
CISP + IN RSV	500	86	414	30	38	18	17.20 ± 1.158 ^**c**^
CISP + IP RSV	500	69	431	34	25	10	13.80 ± 1.068 ^**b**^

### Impact of route of administration of RSV on the histopathological alterations in CISP-intoxicated brains

**Fig. 8:**
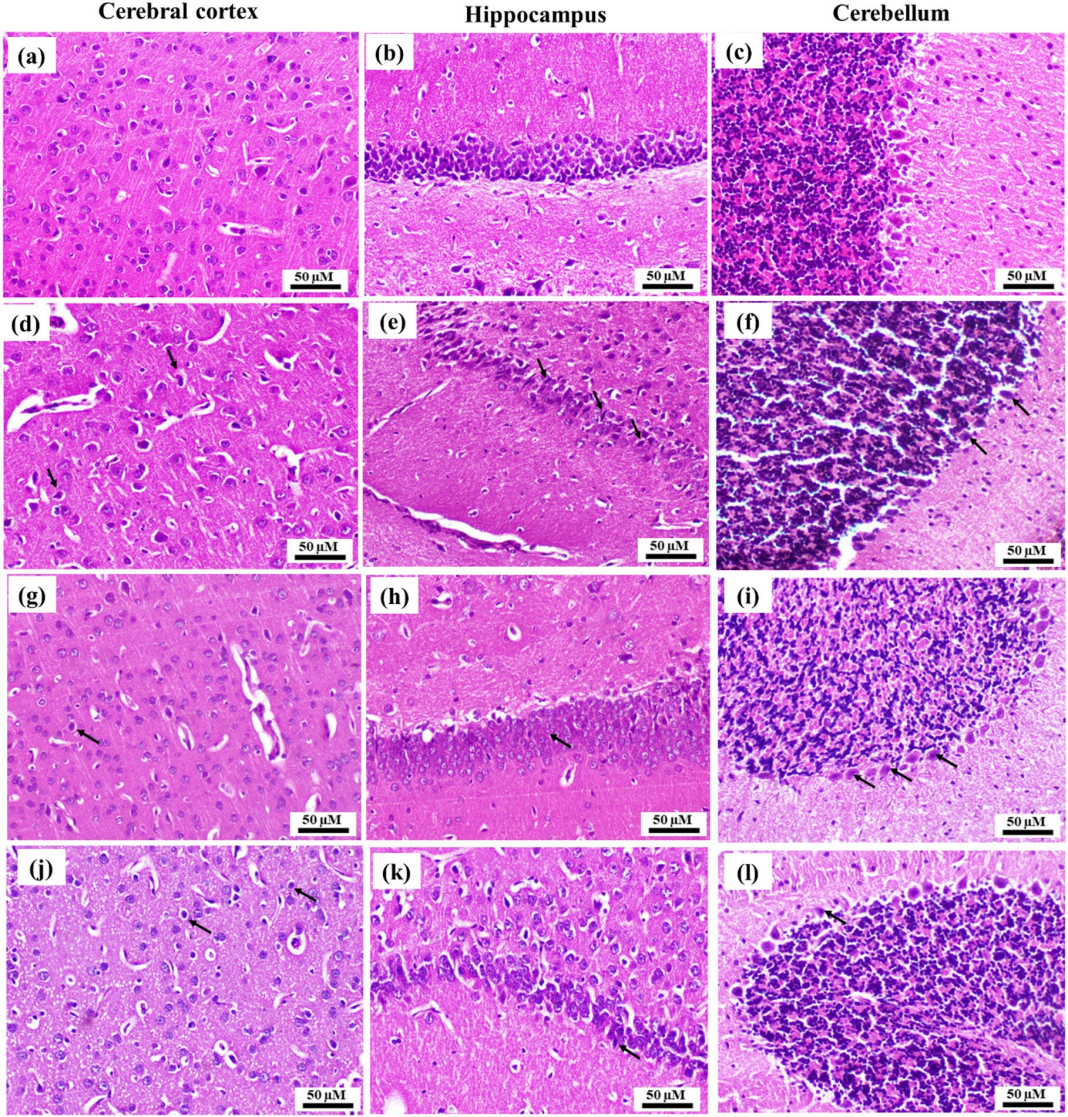
Impact of treatment of CISP-exposed rats with RSV, either IN or IP, on the histopathological alterations in the brain tissues of rats of different groups: representative photomicrographs of different brain regions (cerebral cortices, hippocampi, and cerebella) of different experimental groups: group (1): Negative control (**a**, **b**, and **c**) showing normal histological architecture with intact neurons in the different examined brain regions, group (2): CISP-intoxicated brains (**d**, **e**, and **f**) showing severe neurodegeneration and necrosis of neurons, (d) CISP-neurotoxicated cerebral cortex demonstrating prominent neurodegeneration and necrosis of neurons (arrow); (**e**) CISP-neurotoxicated hippocampus demonstrating obvious pyknosis and shrunken pyramidal neurons (arrow); (**f**) CISP-neurotoxicated cerebellar cortex demonstrating obvious shrunken and pyknotic Purkinje cells (arrow). group (3): CISP + RSV-IN treated brains (**g**, **h**, and **i**) showing moderate neurodegeneration and necrosis of some neurons, (**g**) CISP + RSV-IN treated cerebral cortex demonstrating moderate necrosis of sporadic neurons (arrow); (h) CISP + RSV-IN treated hippocampus demonstrating sparse necrosis of pyramidal neurons (arrow); (**i**) CISP + RSV-IN treated cerebellar cortex demonstrating degeneration of sporadic Purkinje cells (arrow). group 4: CISP + RSV-IP treated brains (**j**, **k**, and **l**) showing mild neurodegeneration necrosis of few neurons; (**j**) CISP + RSV-IP treated cerebral cortex demonstrating necrosis of some neurons (arrow); (**k**) CISP + RSV-IP treated hippocampus demonstrating sparse necrosis of some pyramidal neurons (arrow); (**l**) CISP + RSV-IP treated cerebellar cortex demonstrating mild degeneration of sporadic Purkinje cells (arrow); (scale bar: 50 μm).

Figure 8. and Table 4 illustrated representative photomicrographs of H&E-stained brain regions of:

Negative control rats demonstrating: (a) Negative control cerebral cortex showing normal histological structure, (b) Negative control hippocampus showing normal histological structure, (c) Negative control cerebellum showing normal histological structure (H&EX, 200).

CISP-intoxicated rats demonstrating: (d) CISP-induced cerebral cortex showing neuronal degeneration (arrows) and necrosis of neurons, (e) CISP-induced hippocampus showing neuronal degeneration with decreased neuronal density and marked shrunken and pyknosis of pyramidal neurons in hippocampus, (f) CISP-induced cerebellum showing neurodegeneration and pyknotic and shrunken Purkinje cells (arrows) (H&EX, 200).

Treated CISP + RSV-IN group demonstrating: (g) CISP + RSV-IN group cerebral cortex showing few degenerated neurons (arrow) and necrosis of sporadic neurons, (h) CISP + RSV-IN hippocampus showing nearly normal hippocampus (arrow) and sparse necrosis of pyramidal neurons, (i) CISP + RSV-IN cerebellum showing normal histological structure and intact Purkinje cells (arrows) (H&E, X200).

Treated CISP + RSV-IN group demonstrating: (j) CISP + RSV-IP cerebral cortex showing degeneration of few neurons (arrows) and necrosis of few neurons. (k) CISP + RSV-IP group hippocampus showing few degenerated neurons in hippocampus (arrows) and sparse necrosis of pyramidal neurons. (l) CISP + RSV-IP cerebellum showing few degenerated Purkinje cells (arrows) (H&E, X200).

Our results showed that both IP and IN RSV showed comparable potential to modulate the CISP-induced histopathological alterations in different regions of the brain tissues in rats of different experimental groups.

**Table 4:** Table 4 Impact of treatment of CISP-exposed rats with RSV, either IN or IP, on the histopathological alterations scoring in the brain tissues of rats of different groups. The score system was designed as: score 0 = absence of the lesion in all rats of the group (*n* = 5), score 1= (< 30%), score 2= (< 30% – 50%), score 3= (> 50%).

Histopathological lesions	Negative control	CISP	CISP + RSV-IN	CISP + RSV-IP
Degeneration of neurons in cerebral cortex	0	3	1	1
Degeneration of neurons in hippocampus	0	3	0	1
Degeneration of Purkinje cells in cerebellum	0	3	0	1

### Impact of route of administration of RSV on the histopathological alterations in CISP-intoxicated eyes

**Fig. 9:**
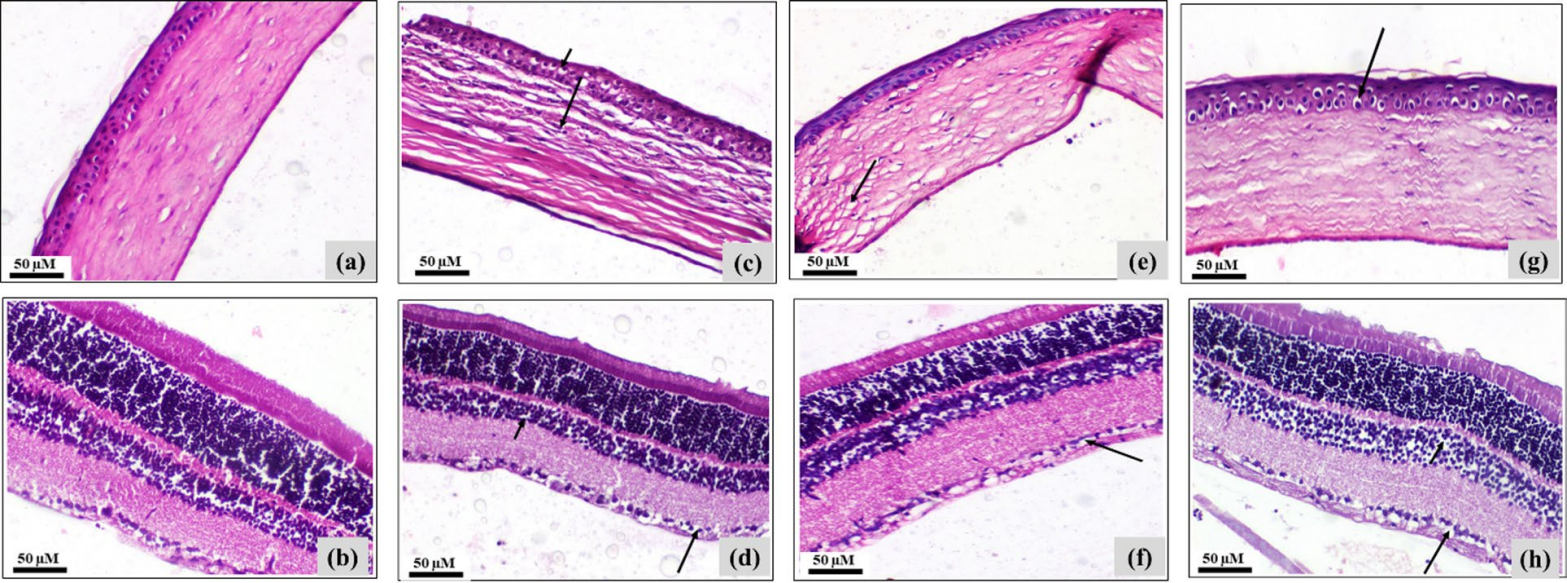
Impact of treatment of CISP-exposed rats with RSV, either IN or IP, on the histopathological alterations in the eye tissues of rats of different groups: representative photomicrographs of H&E-stained corneas and retinas of different experimental groups: group 1: Negative control cornea (**a**) and retina (**b**) normal histological structure, group 2: CISP-exposed cornea (**c**) and retina (**d**) demonstrating edema, inflammatory cells infiltration, and pyknosis, group 3: CISP + RSV-IN treated cornea (**e**) and retina (**f**) showing mild edema and mild vacuolation, respectively, group 4: CISP + RSV-IP treated cornea (**g**) and retina (**h**) showing moderate vacuolar degeneration (scale bar: 50 μm).

Figure 9. and Table 5 illustrated representative photomicrographs of H&E-stained eye regions of:

Negative control rats demonstrating: (a) Negative control cornea showing normal histological structure of cornea, (b) Negative control retina showing normal histological structure of retina (H&E, X200).

CISP-intoxicated rats demonstrating: (c) CISP cornea showing edema and inflammatory cells infiltration in corneal stroma (long arrow) and vacuolation of corneal epithelium (short arrow), (d) CISP retina showing vacuolation of ganglion cell layer (long arrow) and pyknosis in inner nuclear layer (short arrow) (H&EX, 200).

Treated CISP + RSV-IN group demonstrating: (e) CISP + RSV-IN cornea showing mild edema in corneal stroma (arrow), (f) CISP + RSV-IN retina showing mild vacuolation of ganglion cell layer (arrow) (H&E, X200).

Treated CISP + RSV-IN group demonstrating: (g) CISP + RSV-IP cornea showing mild vacuolar degeneration of corneal epithelium (arrow). (h) CISP + RSV-IP retina showing vacuolar degeneration of ganglion cell layer (long arrow) and inner nuclear layer (short arrow) (H&E, X200).

Our results showed that both IP and IN RSV showed comparable potential to modulate the CISP-induced histopathological alterations in different retinal and cortical tissues in rats of different experimental groups.

**Table 5:** Impact of treatment of CISP-exposed rats with RSV, either IN or IP, on the histopathological alterations scoring in the eye tissues of rats of different groups. The score system was designed as: score 0 = absence of the lesion in all rats of the group (*n* = 5), score 1= (< 30%), score 2= (< 30% – 50%), score 3= (> 50%).

Lesions	Control	CISP	CISP+ RSV-IN	CISP + RSV-IP
Vacuolation of ganglion cell layer of retina Pyknosis in inner nuclear layer of retina Edema in corneal stroma Inflammatory cells infiltration in corneal stroma Vacuolar degeneration of corneal epithelium	0 0 0 0 0	3 2 3 2 3	1 0 1 0 0	2 1 0 0 1

## Discussion

The current research aimed at investigating the potential of CISP, as a chemotherapeutic drug, to induce neurotoxicity and neurodegeneration in both the brain and eye tissues of CISP-exposed rats, as well as, comparing the differential neuromodulatory impact of the treatment of CISP-rats with either IN or IP doses of RSV.

IN administration of RSV was used as an alternative route of IP administration, delivering RSV directly to the brain by bypassing the BBB. This research employed the IN administration of RSV as a direct and non-invasive therapeutic approach against CISP-induced neurotoxicity and ocular toxicity in rats. Both IN and IP doses of RSV attenuated oxidative stress and cholinergic dysfunction, mitigated neuroinflammation and apoptosis, and ameliorated the structural dysfunction in both tissues. These therapeutic effects were associated with the upregulation of IL-10 expression, increment of Nrf2 levels, along with downregulation of caspase-8 and DNA damage.

CISP causes DNA damage by its ability to form covalent DNA adducts and monofunctional adducts by binding to a single site on the DNA and forming DNA-protein crosslinks, and thus disrupting the functions of DNA^63^ and proteins^64^. CISP-stimulated DNA damage activates the DNA damage-response pathways, including the p53 signaling axis, driving cell cycle arrest and ultimately enhancing endothelial senescence^25^^,65^^,66^ and activating DNA damage response (DDR) and the subsequent enhancement of various signaling pathways, such as the ataxia telangiectasia mutated (ATM) pathway that lead to cell cycle arrest, DNA repair, or initiation of cell death in case of severe damage^67^^,68^. Herein, we could find that CISP-intoxicated rats exhibited a significant increase in DNA damage % in blood cells by 218.92%, and a significant elevation in 8-OHdG generation in the eye tissues of CISP-rats by 414.39%, as compared to negative control rats. 8-OHdG is has been widely used as a biomarker for oxidative stress: to measure the effect of endogenous oxidative DNA damage^69^.

Oxidative stress is one of the key mechanisms underlying the neurotoxicity of chemotherapy^4^^,70^; this runs in line with our findings that demonstrated that CISP intoxication caused lipid peroxidation and significant decline in CAT activities in both the brain and the eye tissues of CISP-rats, along with significant reduction in the serum Nrf2 levels by -57.85%, as compared to negative control rats, our results run in agreement with previous studies^4^^,71^. This might be ascribed to the high reactivity of CISP that exacerbates oxidative stress in tissues, and inhibited the activity of the anti-oxidant enzymes, resulting in the intracellular accumulation of ROS that can damage cellular structures and exacerbate the senescence phenotype in the BBB and the vascular system of the eye^24,^^70^^,72^. This elevated oxidative stress further destabilizes the endothelial barrier, rendering it more permeable to inflammatory cytokines and neurotoxic molecules^72^.

Our findings run in accordance with a previous study by Altunkaya et al.^26^ that investigated the current dose-based neurotoxic mechanisms of CISP (5, 7.5 and 12 mg/kg) on the hippocampus and cerebral cortex using behavioral, histopathological, and immunohistochemical analyses, and demonstrated that the higher doses of CISP (7.5 and 12 mg/kg) exhibited a significant impact on brain necrosis, apoptosis, and astrogliosis that was manifested as upregulated immune-expression of the apoptotic markers (Bax and Bcl-2) and the neuroinflammation marker (Glial fibrillary acidic protein (GFAP)). This previous study could explain the obvious neurotoxicity in our model that demonstrated significant structural alterations in different brain regions (Fig. 8).

Furthermore, CISP intoxication caused a significant upregulation in the gene expression of caspase-8 by 562%, in CISP-exposed brains, as compared to negative control brains. This runs in good agreement with previous studies that demonstrated that CISP can activate caspases 8, 9, and 3 both in vivo and in vitro^71,73,75^. During the DNA damage process, the superoxide anion (O^2–^), a kind of ROS, is generated that leads to apoptosis^76^. The caspase 8 pathway is activated *via* the TNF-α–caspase 8–caspase 3 axis; caspase 3, the effector of the caspase 8 pathway, may further activate caspase-activated DNases and degrades the regulatory and structural proteins for cell integrity, leading to ROS accumulation and apoptosis^77^.

On the other side, co-treatment of CISP-exposed rats with RSV, either IN or IP significantly mitigated the CISP-induced DNA damage and oxidative status in the same line with a previous study^78^. As compared to CISP-exposed rats, treatment of CISP-rats with either IN or IP RESV demonstrated significant reduction in DNA damage % in blood cells by -27.12 and − 41.53% respectively, in 8-OHdG generation in the ocular tissues by -36.78 and − 47.34%, respectively, in lipid peroxidation (-21.7 and − 16.67%) and (-49.1 and − 40.31%), respectively, in the brain and the eye tissues of CISP-RSV treated rats. While CAT was significantly increased upon treatment of CISP-intoxicated rats with IN or IP RSV (30 and 29.08%) and (10.79 and 26.86%), respectively, in the brain and the eye tissues of CISP-RSV treated rats. Similarly, there was a significant increase in serum Nrf-2 levels by 74.60 and 91.41% respectively for treatment of CISP-rats with IN or IP RSV. In addition, treatment of CISP-rats with RESV either IN or IP demonstrated a significant downregulation in the expression of caspase-8 (-30.66 and 41.69%), as compared to CISP-exposed rats. Collectively, it could be declared that IP RSV demonstrated a superior cumulative DNA protective, and anti-apoptotic potentials, relative to IN RSV.

The anti-oxidative potential of RSV to decrease lipid peroxidation and increase CAT activities, as well as, to activate Nrf2 suggests that the anti-oxidative effect of RSV may be mediated by the mobilization of serum factors. It indicates that the anti-oxidative potential of RSV is involved in regulating blood functions. Our results run in agreement with previous studies of Agcayazi et al.^78^, Zhang et al.^79^, and Pirhan et al.^80^ that demonstrated that the neuroprotective potential of RSV against CISP-induced optic nerve, as well as, its potential of restoring the number of surviving retinal ganglion cells, with riluzole, in a glaucoma-induced experimental model.

Moreover, the anti-oxidative activities of RSV are strongly ascribed to its polyphenolic nature that enabled RSV to scavenge free radical and to modulate Nrf2 activation^81^^,82^; through its potential to regulate cell signaling pathways implicated in inflammation and oxidative stress^83^. RSV also modulated the balance between the oxidant-antioxidant systems by stimulating the Nrf2 signaling pathway^84^, through stimulating phosphoinositide 3-kinase (PI3K)/protein kinase B (Akt) axis, which is crucial for reinforcing antioxidant defense system^85^. Nrf2 regulation is crucial to preserve homeostasis of cellular function^83^. Antioxidants, such as RSV, may help neutralize ROS, thereby preventing oxidative injury and DNA damage and apoptosis, as well as, preserving BBB integrity and vascular integrity of the eye tissues. Furthermore, RSV protected against apoptosis *via* ROS scavenging cerebellar neurons, leading to an increase in cell survival through the Nrf2 pathway^86^; therefore, translating these findings into clinical application could provide a significant progress in treatment of cancer patients, considering the role of Nrf2 in chemoprotection and CISP resistance^87^. Therefore, RSV could be considered Nrf2 activator that could provide protection against CISP- neurotoxicity through decreasing ROS levels and mitigating apoptosis. However, our results run in contrast to other studies who showed that CISP-induced animals exhibited a significantly increased serum Nrf2 levels; due to the over-activation of the Nrf2 pathway to repel oxidative stress and exert a cytoprotective impact to lessen oxidative stress^71^. This could be explained by shorter intoxication time for elaboration of the cytotoxic potential of CISP in the current study. In addition, our findings determined that RSV, either IP or IN, reversed the acute histopathological effects of CISP on the retinal and corneal tissues to a certain degree.

Concerning CISP-induced neuroinflammation, CISP intoxication caused a significant downregulation in the gene expression of IL-10 by -39%, in CISP-exposed brains, as compared to negative control rats. Inflammation can exacerbate tissue injury and impair tissue repair^88^^,89^. Accordingly, the present study investigated whether the anti-inflammatory effects of RSV in the CISP-induced neurotoxicity are attributable to up-regulation of the expression of the anti-inflammatory IL-10. Treatment of CISP-rats with either IN or IP RESV demonstrated a significant upregulation in the expression of IL-10 (286.89 and 363.93%), as compared to CISP-exposed rats; indicating the anti-inflammatory activities of RSV, with a superior activity for IP RSV. A study by Cianciulli et al.^27^ observed that microglia cells submitted to RSV treatment are able to upregulate IL-10 production; due to the potential act of RSV as a polarizing agent in microglia cells, favoring the shifting versus M2 phenotype, more efficient as IL-10 producer. Another possibility is that IL-10 is induced as the predominant effect in these cells, producing an inhibition of pro-inflammatory cytokines in an autocrine/paracrine fashion^90^. These assumptions showed that IL-10 downregulate the release of pro-inflammatory cytokines through decreeing their surface expression receptors^91^^,92^. The anti-nociceptive effect of exogenous administration of IL-10 could be attributed to its capacity to inhibit the release of pro-inflammatory cytokines through suppressing neuroinflammation and microglial activation, furthermore, IL-10 reverses CISP-induced nociceptor hyperactivity and acts directly on sensory neurons^93^. Herein, we propose that RSV administration stimulated the upregulation of IL-10 signaling; therefore, RSV could be developed into a safe therapeutic strategy to prevent or treat chronic chemotherapy-induced neuropathic pain.

In addition, CISP intoxication increased AChE activity; this significant activation of AChE in CISP-exposed rats was previously elaborated^3,^^4^^,71^. Cholinergic dysfunction is a principal sign of neurotoxicity; the significant AChE activation in CISP-intoxicated brains eventually leads to decreased acetylcholine (ACh) release in the synaptic cleft and thus impairs the normal neurotransmission^94^. This elevated AChE activity might be attributed to CISP-induced amplified ROS generation that caused the cholinergic dysfunction. However, a marked decrement in AChE activity was found upon co-treatment of CISP-exposed rats with either IN or IP received RSV by (-42, and − 49.6%, respectively), as compared to CISP-rats; indicating the comparable cholinergic and neuroprotective potential of both IP and IN RSV.

In the current study, we suggested that CISP-treated rats exhibited neurovascular toxicity and endothelial dysfunction as indicated biochemically and histopathologically by cholinergic dysfunction and loss of structural integrity of the brain and eye tissues. Herein, the IN administration of RSV demonstrated comparable neuroprotective activities to those of IP RSV; this could be explained by the potential of IN RSV to avoid the hepatic metabolism, as well as, increased delivery of RSV directly to the brain. Furthermore, RSV can cross the BBB and enter cerebral tissue^95^. Our findings run in agreement with a previous study by Owjfard et al.^96^ that demonstrated that IN administration of RSV showed neurotherapeutic potential and anti-inflammatory activities on experimentally induced ischemic stroke. This highlights the potential therapeutic role of nature-based compounds in alleviating different toxicities in different organs due to their health-promoting potentials^97,99^.

### Study limitations

This study has several limitations that warrant discussion: First, the exclusive use of female rats limits any further analysis of potential sex-specific differences in CISP-induced neurotoxicity or RSV-associated neurotherapeutic activities. Further studies involving male rats are required to investigate the possible hormonal influence on the toxic potential of CISP. Second, the study did not evaluate dose-dependency of CISP; future studies should explore dose-response relationships to provide clinically relevant investigation for optimizing chemotherapy with reduced neurotoxic side effects. Third, the lack of behavioral examination, such as the assessment of cognitive function, to correlate neurotoxic or neuroprotective impacts of either CIS or RSV; involvement of such studies will enable the establishment of a direct relationship between neurotoxicity and cognitive performance in CISP-induced animals.

Our justification to the use of female rats could be explained by the inclusion of CISP in the therapeutic regimens for the most predominant cancers in females; the cervical cancer, ovarian cancer (OC), and breast cancer (BC). CISP, either alone or as an adjuvant or combined with pelvic-radiotherapy, is the basic therapeutic regimen for cervical cancer (the 4th common cancer in females)^100^. In addition, the therapeutic approach for OC, includes the use of CISP, in combination with paclitaxel, with common development of chemoresistance^101^, noting that OC recurs in approximately 80% of the cancer patients with high rates of mortality^102^^,103^. Furthermore, CISP is also used, in combination with doxorubicin, as the first-line anti-neoplastic agent for BC, the most prevalent type of cancer in females above 60 years old^104^.

## Conclusion

In conclusion, IN and IP administration of RSV in a CISP-induced rat model of neurotoxicity and ocular toxicity resulted in significant improvements on the biochemical, molecular, and histopathological levels. Caspase-8 expression was downregulated, while the expression of the anti-inflammatory IL-10 gene was upregulated along with increased serum levels of Nrf2. In addition, RSV mitigated oxidative stress and oxidative DNA damage. IN RSV may be an efficient and non-invasive therapeutic approach for chemotherapy-induced neurotoxicity and further studies are required to confirm its therapeutic potential. This study showed that the anti-oxidative potential of IN RSV was comparable to that of IP RSV; however, the cumulative data indicated that IP RSV was collectively superior to IN RSV by exerting higher anti-apoptotic, anti-cholinergic dysfunction, and anti-inflammatory activities.

There are several future aspects of this study including the use of RSV-loaded nanoparticles that could be administerated through IP or IN routes could present novel and better therapeutic outcomes. The ability to control RSV release through using nanoparicles might improve its therapeutic efficiency. In addition, conducting pharmacokinetis studies will enable the accurate quantification of the pharmacological concentrations of RSV that could reach the brain by both IN or IP routes. Future investigations will involve expanding validation under other pathological conditions and different therapeutic formulations, and including tissue-distribution studies. Moreover, comprehensive research into potential clinical applicability will be essential to integrate novel formulations or dosing methodologies of bioactive molecules into the broader development of therapeutic approaches.

## Data Availability

All data generated or analyzed during this study are included in this published article.
